# The penile cuff test: A clinically useful non-invasive urodynamic investigation to diagnose men with lower urinary tract symptoms

**DOI:** 10.4103/0970-1591.45549

**Published:** 2009

**Authors:** Christopher Harding, Wendy Robson, Michael Drinnan, Stuart McIntosh, Mustafa Sajeel, Clive Giffiths, Robert Pickard

**Affiliations:** 1 Department of Urology, Freeman Hospital, Newcastle upon Tyne, UK; 2 Department of Medical Physics, Freeman Hospital, Newcastle upon Tyne, UK; 3 School of Surgical and Reproductive Sciences, Newcastle University, Newcastle upon Tyne, UK

**Keywords:** Bladder outlet obstruction, non-invasive urodynamics, urinary symptoms

## Abstract

**Objectives:**

To summarize the development of a novel non-invasive test to categorize voiding dysfunction in men complaining of lower urinary tract symptoms (LUTS) - the penile cuff test.

**Methods:**

The test involves the controlled inflation of a penile cuff during micturition to interrupt voiding and hence estimate isovolumetric bladder pressure (p_ves.isv_). The validity, reliability, and clinical usefulness of the test were determined in a number of studies in men with LUTS.

**Results:**

The penile cuff test can be successfully performed in over 90% of men with LUTS. The reading of cuff pressure at flow interruption (p_cuff.int_) gives a valid and reliable estimate of invasively-measured p_ves.isv_ and when combined with the reading for maximum flow rate obtained during the test (Q_max_) produces an accurate categorization of bladder outlet obstruction (BOO). Use of this categorization prior to treatment allows improved prediction of outcome from prostatectomy.

**Conclusion:**

The penile cuff test fulfils the criteria as a useful clinical measurement technique applicable to the diagnosis and treatment planning of men with LUTS.

## INTRODUCTION

Older men frequently complain of troublesome lower urinary tract symptoms typically involving poor urine flow accompanied by day and night-time urinary frequency with an estimated prevalence of 50-60% in men older than 60 years.[1] Urinary symptoms are subjective indicators of possible urinary tract pathology, and in aging men the predominant cause is thought to be prostatic enlargement leading to obstruction of the bladder outlet. transurethral prostatectomy (TURP) is an operation designed to alleviate lower urinary tract symptoms (LUTS) secondary to bladder outlet obstruction (BOO) but not all men with LUTS will suffer from BOO and a fair proportion will not benefit from this type of surgery. Recent classification has emphasized the differences between;

- LUTS, which can be caused by a host of urinary tract pathologies other than prostate disease.
- Clinical benign prostatic enlargement (BPE), which becomes more common with increasing age and is secondary to histological benign prostatic hyperplasia (BPH).
- BOO which can only be currently diagnosed from pressure-flow analysis of voiding.

The relationship of LUTS, BPE, and BOO is complex and the presence of one does not necessarily imply the presence of others. LUTS are assessed via symptom and quality of life questionnaires and although these tools are well-validated measures of symptom severity and bother they are of limited diagnostic or prognostic use. This is because LUTS are entirely subjective and in general not sufficiently disease-specific to allow a clinical diagnosis to be made. BPE is a clinical diagnosis that is increasingly common with advancing age but may not result in symptoms or affect quality of life. The cause of BPE is invariably BPH but this is a histological diagnosis that can only be made by pathological examination of prostate tissue. BOO is characterized by high voiding pressures and a reduced urine flow rate but is not exclusively secondary to BPE. To make the diagnosis of BOO invasive pressure-flow studies (PFS) must be performed but this investigation is time-consuming, requires expensive equipment and skilled staff and carries a recognized risk of urinary tract infection (UTI). For these reasons PFS are not routinely used in the investigation of patients with LUTS and are often reserved for selected cases.[2]

## THE PENILE CUFF TEST

**Figure 1:**
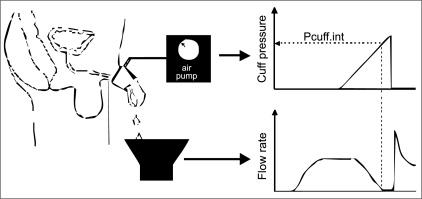
Diagrammatic representation of the penile cuff test. Cuff inflation continues until flow ceases or a safety cut-off of 200 cmH_2_O is reached. Following flow arrest, the cuff is deflated producing the characteristic surge in flow rate.

As a result of these drawbacks to invasive PFS, interest has focused on the development of non-invasive methods to diagnose BOO.[3] One such technique is the penile cuff test, which involves inflation of a pneumatic cuff placed around the penis to interrupt the urinary stream. The technique was first described experimentally in baboons[4] and then later applied in humans.[5] In Newcastle we have further developed the technique with the cuff being inflated during established voiding until urine flow is interrupted [Figure 1].

At flow interruption, the resultant continuous column of fluid from urethra to bladder acts as a manometer giving an indirect measurement of bladder pressure using a principle similar to that of systolic blood pressure measurement with the cuff pressure at interruption of flow (p_cuff.int_) being theoretically equal to isovolumetric bladder pressure. The cuff then deflates, flow resumes and the cycle can begin again allowing multiple inflation cycles during a single void. For safety if the urinary stream is not interrupted before the cuff pressure reaches 200 cmH_2_O then deflation will occur automatically. Device development has allowed full automation using a custom-made cuff inflation device and standard PC (CT3000; Mediplus Ltd, High Wycombe, UK). The test is combined with a urine flow rate measurement using a linked flow meter to provide combined pressure and flow measurements during a single void.

## METHODS (1)

### Clinical validation of basic principles

If cuff pressure at flow interruption (p_cuff.int_) is to reflect isovolumetric bladder pressure (p_ves.isv_) the following conditions must be met: -

#### 1. Cuff pressure is transmitted to the urethral lumen

Thirty patients and five volunteers underwent simultaneous recording of penile cuff pressure and direct measurement of urethral pressure during an inflation-deflation cycle. Excellent agreement was found over the pressure range 0 to 200 cmH_2_O and the results were shown to have good within-subject repeatability.[6] For a fixed cuff pressure of 120 cmH_2_O the mean (SD) urethral pressure was found to be 118 (16.3) cmH_2_O.

#### 2. Bladder pressure is transmitted to the urethra

The hypothesis that pressures are equal throughout the urethra and bladder proximal to the occluding cuff was tested in a study involving 11 men.[7] Simultaneous invasive PFS and penile cuff test were performed with the addition of a transducer-tipped catheter placed in the urethra proximal to the cuff. This allowed the direct measurement of both bladder and proximal urethral pressure during cuff inflation and subsequent flow arrest. Results showed excellent agreement between bladder pressure and urethral pressure measured directly at the time of flow interruption confirming that p_cuff.int_ estimates p_ves.isv_.

#### 3. Bladder contraction is maintained following arrest of flow

To determine whether bladder contraction was maintained despite interruption of voiding by the inflated cuff data from 26 men with LUTS and 5 healthy volunteers underwent combined invasive PFS and penile cuff test[8] recording bladder pressure before, during and after flow int erruption. As expected bladder pressure increased following imposition of isovolumetric conditions whilst bladder pressure before and after the cuff inflation cycle were similar with a mean difference of <5 cmH_2_O showing that bladder contraction was maintained during cuff inflation and subsequent flow interruption.

### Initial clinical evaluation

Confirmation that these basic assumptions were correct allowed us to proceed to a pilot study to assess the accuracy of p_cuff.int_ as a non-invasive estimate of p_ves.isv_.[9] 32 men with LUTS and 7 volunteers underwent simultaneous invasive PFS and penile cuff testing. Good agreement was found between cuff pressure at the time of flow arrest (p_cuff.int_) and p_ves.isv_. It was found that p_cuff.int_ overestimated p_ves.isv_ by a mean (SD) of 14.5 (14) cmH_2_O. This discrepancy was partly explained by the height difference in reference points for the two measurements whereby the anterior edge of the symphysis pubis is used for invasive PFS compared to the mid-penile urethra, approximately 10 cm lower, for the cuff test. These positive findings led us to perform a large-scale clinical evaluation of the technique.

### Results (1)

Consideration of the preliminary clinical studies enabled us to set a series of rules to decide whether a particular test should be accepted as a measurement or repeated.[10]

- No recovery of urine flow following cuff deflation suggests that voiding has finished during the preceding cuff inflation and the recorded pressure should not be used for clinical measurement.
- Erratic flow during cuff inflation leads to ambiguity regarding measurement of p_cuff.int_ and the test should be repeated avoiding abdominal straining.
- All inflation-deflation cycles obtained during a single void should be considered and the maximum value for p_cuff.int_ used as long as it is consistent with other pressure readings obtained during the same test.

## METHODS (2)

### Large scale clinical study

**Figure 2:**
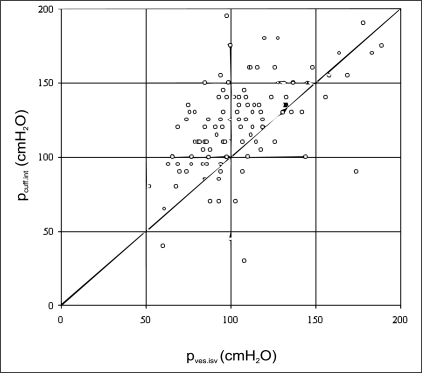
Comparison of cuff interruption pressure and directly measured isovolumetric bladder pressure in a large-scale clinical evaluation illustrating the overestimation of approximately 15 cmH_2_O. Each point represents readings of isovolumetric bladder pressure (p_ves.isv_) and cuff pressure at interruption of flow (p_cuff.int_) measured simultaneously in 151 men with LUTS

A clinical study was undertaken to assess test applicability to men with LUTS.[11] 151 symptomatic men underwent simultaneous invasive cystometry and penile cuff test to assess the accuracy of cuff interruption pressure as an estimate of isovolumetric bladder pressure. For the 117 (77%) subjects providing data acceptable for evaluation, p_cuff.int_ overestimated p_ves.isv_ by a mean (SD) of 16.4 (27.5) cmH_2_O with the discrepancy again mainly due to the height difference in reference point [Figure 2]. This was confirmation that the penile cuff test maintained acceptable levels of accuracy within a large-scale clinical setting.

### Development of the non-invasive nomogram

Objective classification of BOO in men with LUTS is currently based on measurements made during invasive PFS and whilst this improves prediction of good outcome from TURP it is limited by the time consuming nature, expense, and risk of UTI associated with conventional cystometry.[2] We therefore determined whether measurements obtained during the penile cuff test could be used to classify outlet status and guide treatment decisions for men with LUTS.

Classification of obstruction using invasive PFS measurements is usually based on plotting simultaneous measurements of detrusor pressure at maximum flow rate (p_det.Qmax_) and maximum flow rate (Q_max_) on a pressure-flow diagram with the provisional ICS nomogram now the recommended method.[12] The pressure reading obtained during the penile cuff test (p_cuff.int_) differs from p_det.Qmax_ in two important ways: First, p_cuff.int_ includes abdominal pressure (p_abd_) and, second, p_cuff.int_ estimates isovolumetric bladder pressure (p_ves.isv_) since flow = 0 at the time of measurement. To adjust for these differences the intercept of the division line separating obstructed from equivocal/unobstructed groups with the vertical axis has to be increased from 40 to 80 cmH_2_O which reflects the contribution of abdominal pressure in the standing position (35 cmH_2_O) and the lower reference point for pressure measurement used in the cuff test (8 cm).[13] The slope of the line then has to be increased from the original value of 2 cmH_2_O per ml/s to 4 cmH_2_O per ml/s to allow for the pressure rise under isovolumetric conditions.[7] The resultant non-invasive pressure-flow nomogram constructed by these adjustments was then prospectively validated.[14]

### Results (2)

**Figure 3:**
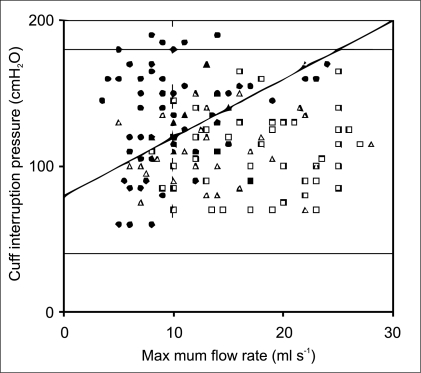
Pressure (p_cuff.int_) and urine flow (Q_max_) readings obtained using the penile cuff test from 144 men with LUTS plotted on the proposed non-invasive nomogram. Symbols represent categorization using conventional invasive PFS (· = obstructed, Δ = equivocal,  = unobstructed). The non-invasive nomogram is divided into 4 quadrants by an oblique line (pcuffint = 80 + 4Q_max_) and a vertical line (Q_max_ = 10) to categorize individual plots as obstructed (upper left), non obstructed (lower right) or diagnosis uncertain (lower left and upper right).

In 144 men from 2 clinical centers readings for detrusor pressure at maximum flow (pdet.Q_max_) and maximum flow rate (Q_max_) were obtained from conventional PFS and plotted on the ICS nomogram for classification of obstruction. A non-invasive penile cuff test was then performed. For each patient, p_cuff.int_ was plotted against Q_max_ on the modified nomogram, using a symbol to indicate his or her standard classification from invasive PFS. Figure 3 illustrates the result of plotting the data on the modified nomogram. For patients above the proposed obstructed line the positive predictive value (PPV) for obstruction was 68% and sensitivity 64%.[14] For patients below the line the negative predictive value (NPV) for equivocal/unobstructed was 78% and specificity 81%. The chi-squared test demonstrated that it was extremely unlikely the proportion of unobstructed above the line occurred by chance (χ^2^ = 29.8; *P* < 0.001).

Applying the commonly used criterion of Q_max_ <10 ml/s alone as a predictor of obstruction we found: PPV 77%, sensitivity 59%, NPV 77%, specificity 89%. However, for the 68% of patients where both the pressure and the flow criteria agreed either the patient was obstructed or the patient was equivocal/unobstructed (PPV 88%, NPV 86%). The detection of obstruction by both the modified nomogram and the flow rate criterion of <10 ml/s are similar confirming that both techniques provide clinically useful data. There is a clear further improvement in predictive value for the 68% of patients when both methods are in agreement: either indicating obstruction (PPV 85%) or indicating equivocal/unobstructed (NPV 90%). These accuracy rates indicate that the classification using both the pressure and flow criteria is clinically useful and suggest that the new technique, in addition to flow rate alone, can play a useful role in the management of patients with LUTS.[14]

## METHODS (3)

### Prediction of outcome from TURP

As detailed above the penile cuff test provides useful data for diagnosing BOO using the proposed non-invasive nomogram. There is evidence from several studies that men with BOO will benefit most from surgical treatment such as TURP with surgical success rates of up to 90% in those with BOO categorized by invasive PFS compared to 70-75% in men who did not undergo invasive PFS pre-operative and were selected for surgery on flow rate and symptoms alone.[2] The next stage in the evaluation of the clinical usefulness of the penile cuff test was to investigate the predictive value of categorization of BOO using the non-invasive pressure and flow measurements in the context of outcome from TURP. In addition, by repeating the cuff test 4 months after TURP we assessed whether non-invasive pressure measurement was sensitive to change following removal of obstruction.

We recruited 208 men for this study who had been selected to undergo TURP on the basis of a reduced flow rate and bothersome symptoms affecting their well-being with only 20% having had invasive PFS.[15] A total of 179 (86%) men provided data suitable for analysis whilst of the 29 men excluded, the test failed in 15 (7%), 9 (4%) did not undergo TURP, and 5 were lost to follow-up. The median (range) age of the patients was 68 (47-88) years and the median (range) time to post-operative assessment was 19 (9-29) weeks. The overall surgical success rate was 77%, slightly higher than in previous series using similar selection criteria.[16]

### Results (3)

Values of p_cuff.int_ and Q_max_ obtained during the preoperative penile cuff test were used to categorize each patient according to the proposed non-invasive nomogram.[14] The results showed that 71 men were categorized as obstructed, 36 as not-obstructed and in 72 the diagnosis was uncertain. A definitive urodynamic diagnosis of BOO/no BOO was therefore achieved for 107 (60%) men using the non-invasive penile cuff test.

The accuracy of prediction of a satisfactory surgical outcome from TURP of these diagnostic categories was examined by comparing the relative surgical success rates for each group. The mean (95% CI) surgical success rate for men categorized as obstructed using the non-invasive penile cuff test was significantly higher at 87% (79-95%) compared to 56% (40-72%) in those classified as not obstructed (P=0.001) and 78% (68-88%) in the group classified as diagnostically uncertain [Figure 3].

**Figure 4:**
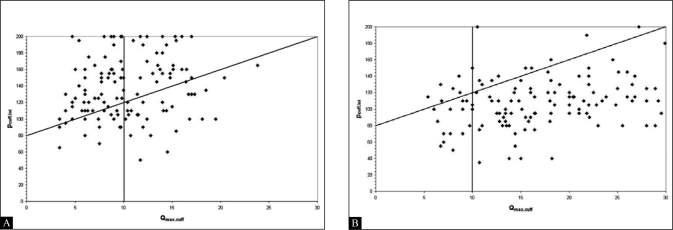
Bladder pressure and urine flow readings obtained with the non-invasive penile cuff test from 149 prior to TURP (A) and 4 months following TURP (B) plotted on the non-invasive nomogram. A marked movement of individual plots from the upper left obstructed area of the nomogram to the lower right not obstructed area is seen.

[Table 1] details the pre and post-operative diagnostic categories of the 143 (69%) men who underwent penile cuff testing both before and after surgery. These data revealed that following TURP most patients (79%) are classified as not obstructed and very few (4%) remain classified as obstructed after surgery.[17] This change in categorization resulted from both an expected increase in mean (SD) Q_max_ from 11 (4) ml/s to 19 (8) ml/s but also a substantial and statistically significant fall in mean (SD) isovolumetric bladder pressure measured by p_cuff.int_ from 138 (35) cmH_2_O to 110 (29) cmH_2_O (*P*<0.0001) suggesting altered detrusor contractility [Figure 4].

**Table 1:** Pre and postoperative non-invasive urodynamic diagnosis in 143 patients who underwent a penile cuff test tested before and 4 months after TURP. The majority of patients as expected change category to not obstructed following TURP.

	Categorization after TURP		
	
Categorization before TURP	Obstructed	Uncertain	Not obstructed
Obstructed (*n*=59)	4	11	44
Uncertain (*n*=50)	2	13	35
Not Obstructed (*n*=34)	0	0	34

Overall, this study illustrates the large-scale application of the penile cuff test to men undergoing endoscopic prostatectomy. The majority of recruited patients (93%) were able to undergo the test successfully and provide measurements suitable for categorization of obstruction.

### Comment

Taken as a whole, the results of these studies show that an accurate non-invasive categorization of obstruction is possible for about 60% of men with LUTS and those men classified as obstructed have an enhanced likelihood of a good outcome from surgical treatment. It should be noted that the penile cuff test combines a measure of contraction strength, isovolumetric bladder pressure, and a measure of outlet resistance, urinary flow rate and the encouraging results from using this combination for clinical purposes may be due to the fact that although other conditions can share either increased contractility or diminished flow those with bladder outlet obstruction are more likely to exhibit both. Increased levels of contractility may be associated with conditions such as detrusor overactivity and these patients will usually have normal or high urine flow rates placing them in the upper right quadrant of the non-invasive nomogram. In addition, diminished flow alone can be secondary to detrusor hypocontractility but in these cases by definition contraction strength is reduced and they are likely to be plotted in the lower left quadrant. In common with previous studies using invasive data,[18] our results show that surgical success rate from TURP is maximal in patients with high levels of contraction strength and low maximum urine flow rates whose penile cuff test measurements place in the upper left quadrant of the non-invasive nomogram and a success rates of close to 90% would be expected.[15,18]

Both increased contraction strength measured by invasive cystometry and diminished urine flow rate have previously been shown to change following TURP[19] and the changes in categorization by the non-invasive nomogram are consistent with these findings.[17] The majority of patients (75%) who were preoperatively diagnosed as obstructed using the penile cuff test became not obstructed after surgery and reassuringly all 34 patients who were not obstructed according to the proposed non-invasive nomogram remained so after TURP. These findings provide further evidence to validate the clinical usefulness of measurements made by the penile cuff test.

## CONCLUSION

Use of the penile cuff test and proposed non-invasive nomogram appear helpful in the evaluation of men with LUTS. It provides a means of obtaining a urodynamic diagnosis with a high level of accuracy without the morbidity and expense of invasive PFS. The cuff test may be particularly useful in the counseling of patients prior to TURP. Patients diagnosed as obstructed following a penile cuff test can be reassured that surgery has a high chance of resulting in symptomatic benefit [Figure 5]. Furthermore, those patients who undergo a penile cuff test and are diagnosed as not obstructed may wish to try other non-surgical treatment options in the light of the poor surgical outcome in this group.

**Figure 5:**
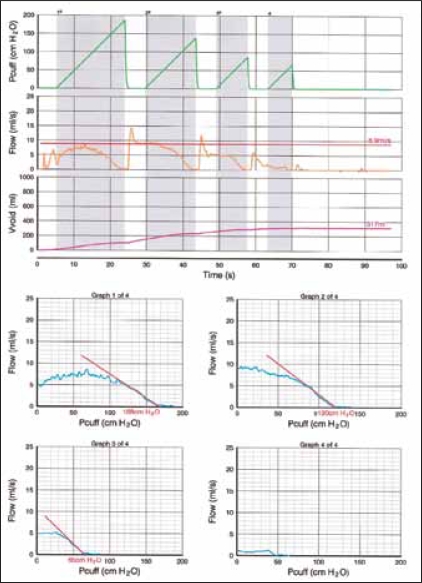
Reproduction of output from an individual penile cuff test showing the pressure flow plot (A) and the resultant readings plotted on the non-invasive nomogram

**Figure 5B:**
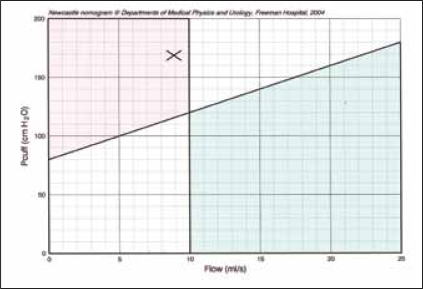
(B) categorizing the void as obstructed
